# Interleukin-6 Promotes Epithelial-Mesenchymal Transition and Cell Invasion through Integrin *β*6 Upregulation in Colorectal Cancer

**DOI:** 10.1155/2020/8032187

**Published:** 2020-08-13

**Authors:** Qi Sun, Yukui Shang, Fengkai Sun, Xiwen Dong, Jun Niu, Fanni Li

**Affiliations:** ^1^Department of General Surgery, The First Affiliated Hospital of Xi'an Jiaotong University, Xi'an 710061, China; ^2^Department of Basic Medical Sciences, School of Medicine, Tsinghua University, Beijing 100084, China; ^3^Department of Gastroenterology, Shandong Provincial Hospital Affiliated to Shandong First Medical University, Jinan 250021, China; ^4^Department of Experimental Hematology, Beijing Institute of Radiation Medicine, Beijing 100850, China; ^5^Department of General Surgery, Qilu Hospital of Shandong University, Jinan 250012, China; ^6^Department of Talent Highland, The First Affiliated Hospital of Xi'an Jiaotong University, Xi'an 710061, China

## Abstract

The metastatic potential of colorectal cancer (CRC) is intensively promoted by the tumor microenvironment (TME) in a paracrine manner. As a pleiotropic inflammatory cytokine, Interleukin-6 (IL-6) is produced and involved in CRC, the same scenario where integrin *α*v*β*6 also becomes upregulated. However, the relationship between IL-6 and integrin *α*v*β*6 as well as their involvement in the crosstalk between CRC and TME remains largely unclear. In the present study, we demonstrated a positive correlation between the expression of IL-6 and integrin *β*6 in CRC samples. The mutually promotive interaction between CRC and TME was further determined by an indirect coculture system. CRC cells could augment the secretion of IL-6 from fibroblasts, which in return induced invasion and integrin *β*6 expression of CRC cells. Through the classic IL-6 receptor/STAT-3 signaling pathway, IL-6 mediated the upregulation of integrin *β*6, which was involved in the invasion and epithelial-mesenchymal transition of CRC cells induced by IL-6. Taken together, our results reveal a paracrine crosstalk between IL-6 signals originating from the TME and increased the integrin *β*6 level of CRC. IL-6 induces CRC invasion via upregulation of integrin *β*6 through the IL-6 receptor/STAT-3 signaling pathway. Combined inhibition of IL-6 along with integrin *β*6-targeted strategy may indicate new directions for antitumor strategies for CRC.

## 1. Introduction

Due to environmental causes and risk factors in modern society such as intestinal pathogens and inflammation, colorectal cancer (CRC) is among the most common malignancies [1]. Distant metastasis remains one of the leading contributors of CRC-related deaths, despite recent advancements in surgery techniques and adjuvant therapy. Therefore, a better understanding is urgently needed to investigate the mechanism of CRC metastasis so as to further improve the clinical outcomes.

Even though many vital molecular mechanisms have been revealed involved in cancer progression, it seems not realistic to prevent CRC metastasis by targeting one oncogene or signaling pathway within cancer cells [2]. The tumor consists of malignant cancer cells and stromal cells that constitute the tumor microenvironment (TME). More and more studies have discovered that the metastatic potential of cancer cells not only depends on certain oncogenes and signaling pathways but also is intensively promoted by the TME in a paracrine manner [3]. The stromal cells in the TME can be activated by cancer cells but meanwhile promote the epithelial-mesenchymal transition (EMT) of cancer cells the other way round through secretion of various factors including chemokines and cytokines [4]. Interleukin-6 (IL-6), a pleiotropic inflammatory cytokine which plays a vital role in immune and inflammatory response, is as well considered as a key growth factor for malignancy. IL-6 can be produced by multiple cell types located within the TME, including tumor-infiltrating immune cells, stromal cells, and cancer cells themselves [5]. During classic signaling, IL-6 binds to IL-6 receptor (IL-6R), forming the heterohexameric complex followed by recruitment of cellular proteins and subsequent activation of signaling pathways including JAK/STAT-3, PI3K, and MAPK [6]. IL-6 has been shown to increase metastatic capability in a variety of tumor models by multiple mechanisms. IL-6 promotes cancer stemness and oncogenicity of osteosarcoma cells by upregulating a secreted phosphorylated glycoprotein osteopontin [7]. In addition to tumorigenesis, IL-6 contributes to chemoresistance and recurrence [8]. Moreover, IL-6 can serve as a prognostic indicator of poor survival [9, 10] as well as a predictor of therapeutic response [11, 12] in a wide range of tumors.

The best described mechanism under which IL-6 increases tumor invasion is by conferring tumor cells on an EMT phenotype, through which tumor cells transit from adherent epithelial to mobile mesenchymal states thus facilitating invasion and metastasis [13]. During the complex process of metastasis, integrins, a large family of cellular adhesion receptors, are involved in almost every step by mediating the adhesion and interaction of the invading cancer cells with surrounding cells and extracellular matrix (ECM) [14]. So far, 24 different subtypes of integrins, composed of *α* and *β* subunits, have been identified [13]. Integrin *α*v*β*6 is the only subtype the *β*6 subunit could constitute, which is specifically expressed in the epithelia. Integrin *α*v*β*6 is barely expressed in adult normal tissues but becomes substantially upregulated in the context of inflammation and malignancy, acting as an indicator of tumor progression [15]. Our previous researches have defined the important participation of integrin *α*v*β*6 in cancer invasion, matrix metalloproteinase (MMP) secretion, and chemoresistance [16–18]. In addition, integrin *α*v*β*6 plays a bidirectional regulation role between CRC cells and stromal cells [19].

Previous studies have reported that IL-6 associates with CRC progression. IL-6 is not only related to advanced tumor stages and poor survival of CRC patients [20] but also promotes the motility of CRC cells [21]. IL-6 is produced and involved in inflammation and carcinogenesis, the same scenario where integrin *α*v*β*6 also becomes upregulated. A correlation of expression of IL-6 with integrin *α*v*β*6 was recently reported [22]; however, many questions still remain as to whether the expression of integrin *α*v*β*6 is mediated by the activation of the IL-6 signaling pathway rather than being a concomitant phenomenon, since IL-6 may directly induce EMT of cancer cells to promote metastasis. Furthermore, the downstream mechanism of IL-6-induced invasion as well as the relationship between IL-6 and integrin *α*v*β*6 remains largely unknown. In this study, we analyzed the effect of IL-6 on the expression of integrin *α*v*β*6 and further characterized the roles of IL-6 and integrin *α*v*β*6 in the paracrine crosstalk between the TME and CRC cells.

## 2. Materials and Methods

### 2.1. Cell Culture and Transfection

CRC cell lines HT-29 and Colo205 and fibroblast cell line CCD-18Co from the colon were purchased from ATCC. Human normal colon mucosal epithelial cell line NCM460 was obtained from INCELL. HT-29 and Colo205 were cultured in complete Dulbecco's modified Eagle's medium (DMEM) containing 10% fetal bovine serum (FBS), with essential supplementation including penicillin/streptomycin solution, while NCM460 in complete RPMI-1640 medium. CCD-18Co was cultured in ATCC-formulated Eagle's Minimum Essential Medium (#30-2003) with 10% FBS. All the cells were incubated at 37°C in a 5% CO_2_ humidity atmosphere. The recombinant human IL-6 was purchased from R&D Systems. For the function blocking, the specific antibody 10D5 against integrin *β*6 was purchased from Merck Millipore and neutralizing anti-IL-6 as well as anti-IL-6R from R&D Systems. To knock down integrin *β*6 (#144659), IL-6R (#106147), or STAT-3 (#116558), cells were transfected with specific siRNA (Thermo Fisher Scientific) using Lipofectamine 3000 (Invitrogen) according to the manufacturer's instructions. For the inhibition of the STAT-3 pathway, specific STAT-3 inhibitors Stattic and Cryptotanshinone (CTS), ERK/MAPK inhibitor U0126, and PI3K inhibitor LY294002 were purchased from SelleckChem.

### 2.2. Primary CRC Samples

For the immunohistochemistry (IHC) assay, a total of 155 CRC patients with intact clinical information who underwent radical operation were selected between May 2015 and June 2017 from the First Affiliated Hospital of Xi'an Jiaotong University. All the samples were histopathologically confirmed by two pathologists independently. Written informed consents were obtained from all patients. The protocol was approved by the Institutional Medical Ethics Committee of the First Affiliated Hospital of Xi'an Jiaotong University.

### 2.3. IHC

IHC staining was performed on formalin-fixed paraffin-embedded CRC slide sections. Optimal antigen retrieval was induced in citrate buffer at pH 6.0 and 95°C for 30 min. The sections were blocked with 10% normal goat serum, followed by incubation with primary antibody against IL-6 (#6672, Abcam) or integrin *β*6 (Clone 442.5C4, Millipore Sigma) overnight at 4°C before incubation with secondary antibody conjugated with HRP. Finally, the staining was established by a DAB peroxidase substrate (Beyotime). The extent and intensity of IHC staining for IL-6 and integrin *β*6 were scored by two blinded observers under a light microscope. Briefly, the intensity of staining was graded as 0 (negative), 1 (weak), 2 (moderate), or 3 (strong) and the extent of staining as 0 (0%), 1 (1-25%), 2 (26-50%), 3 (51-75%), or 4 (76-100%). The product of intensity and extent was used as the final IHC score. The patients were further classified into low or high expression group based on IL-6 immunostaining with the median IHC score as the cut-off value.

### 2.4. Coculture Assay

The indirect coculture of CRC cells with fibroblasts was established using Transwell membranes (0.4 *μ*m pore size, Merck Millipore) for the paracrine crosstalk study. CCD-18Co cells (5.0 × 10^4^ cells) were seeded on the bottom of a 24-well culture plate with 500 *μ*l fibroblast culture medium. In addition, HT-29 or Colo205 cells (1.0 × 10^5^) were seeded onto Transwell inserts with 100 *μ*l DMEM. After 12 h, the Transwell inserts with cancer cells were transferred to the culture plate containing fibroblasts and cocultured for 72 h. As a blank control (monoculture), fibroblast culture medium without CCD-18Co in the plate or DMEM without cancer cells in the Transwell inserts was prepared.

### 2.5. Enzyme-Linked Immunosorbent Assay (ELISA)

The culture medium was collected after coculture for 72 h and centrifuged to detect the concentration of supernatant IL-6 using the human IL-6 ELISA kit (Boster) according to the manufacturer's instructions.

### 2.6. Quantitative PCR (qPCR)

Total RNA of cells was harvested using the TRIzol reagent, and cDNA was synthesized following the manufacturer's protocol (TaKaRa). qPCR was performed on a LightCycler 480 system (Roche Life Science). For the calculation of the fold change of the mRNA levels, the 2^-*ΔΔ*Cq^ method was used with the human housekeeper gene GAPDH as the internal reference. The primers of human integrin *β*6 were forward 5′-TCCATCTGGAGTTGGCGAAAG-3′ and reverse 5′-TCTGTCTGCCTACACTGAGAG-3′. The primers of human integrin *α*v were forward 5′-GGCTGCATATTTCGGATTTTCTG-3′ and reverse 5′-CCATTCAGCTTTGTCGTCTGG-3′. The primers of human GAPDH were forward 5′-CTGGGCTACACTGAGCACC-3′ and reverse 5′-AAGTGGTCGTTGAGGGCAATG-3′.

### 2.7. Western Blotting

The protein lysates were harvested using RIPA lysis buffer, with quantification performed using the Pierce BCA kit (Thermo Fisher Scientific). For immunoblot analysis, 25-50 *μ*g protein was boiled in Laemmli buffer and loaded on an 8-10% denaturing SDS-polyacrylamide gel and then transferred to PVDF membranes, which were blocked with 5% bovine serum albumin before immunoblotting with primary antibodies against IL-6R (#167742, Abcam), integrin *β*6 (Clone 442.5C4, Millipore Sigma), phospho-STAT-3 (#9145), total STAT-3 (#4904), phospho-ERK1/2 (#9106), phospho-AKT (#4060), Vimentin (#5741), E-cadherin (#3195) (Cell Signaling Technology), *α*v (#sc-9969), and *β*-actin (#sc-47778) (Santa Cruz). After the incubation with secondary antibodies, the bands were detected by enhanced chemiluminescence.

### 2.8. Transwell Invasion Assay

The invasion capability of cells was assessed using Transwell Boyden chambers (Corning, 8 *μ*m pore size) precoated with Matrigel. After treatment with the indicated reagent or siRNA, 1.0 × 10^5^ CRC cells in serum-free medium were seeded on the upper chamber, with medium containing indicated doses of IL-6 in the lower chamber for 24 h. For the coculture Transwell invasion assay, fibroblasts (1.0 × 10^5^) were seeded to the lower chamber in 600 *μ*l culture medium, and CRC cells (1.0 × 10^5^) were seeded on the upper chamber in 100 *μ*l serum-free DMEM with a coculture interval of 24 h. The monoculture system without fibroblasts was used as the control. After the removal of the cells on the upper side of the membrane, the cells on the lower surface were fixed, stained, and counted in four random fields (magnification, ×400).

### 2.9. Statistics

Statistical analyses were performed using GraphPad Prism 5 software. Data were presented as means ± SEM. Student's *t*-tests were performed for the comparison of two means while ANOVA for 3 or more data sets. The association between IHC staining of IL-6 and integrin *β*6 was determined by the Mann-Whitney test. Chi-squared tests were used to explore the association of IL-6 and integrin *β*6 expression with different clinicopathological variables. *p* < 0.05 was considered to be statistically significant.

## 3. Results

### 3.1. There Is a Positive Correlation between the Expression of IL-6 and Integrin *β*6 in CRC Samples

**Figure 1:**
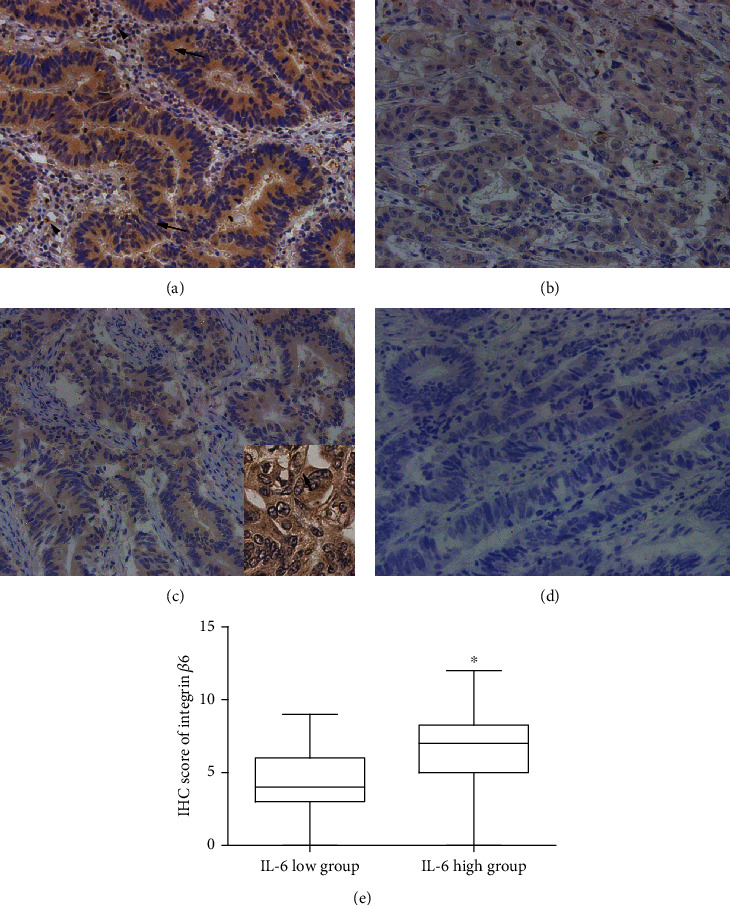
Immunostaining of IL-6 and integrin *β*6 in CRC samples (magnification, 400x). (a) High expression of IL-6 in the tumor (arrows) as well as stromal cells (arrowheads). (b) Low expression of IL-6. (c) Positive expression of integrin *β*6 in the tumor cells (arrow in the enlargement). (d) Negative expression of integrin *β*6. (e) Box-and-whisker plot of IHC score indicates higher immunostaining of integrin *β*6 in the IL-6-high group than in the IL-6-low group. ^∗^*p* < 0.05. IHC: immunohistochemistry.

IHC analysis demonstrated that the immunostaining of IL-6 was detected predominantly in the cytoplasm of tumor as well as stromal cells (Figures 1(a) and 1(b)) while the staining of integrin *β*6 in the cytoplasm and membrane of tumor cells (Figures 1(c) and 1(d)). Among the 155 CRC samples, 76 cases (49%) showed high IL-6 expression and 61 cases (39%) showed positive expression of integrin *β*6. To further investigate whether there was a correlation between IL-6 and *β*6 expression, the samples were divided into high and low expression groups based on IL-6 immunostaining, and the IHC scores of integrin *β*6 from two groups were compared by Mann-Whitney analysis, which indicated that the IL-6-high group demonstrated higher immunostaining of integrin *β*6 than the IL-6-low group (Figure 1(e)). Therefore, the expression of IL-6 was positively related to the expression of integrin *β*6 in CRC samples. The association of IL-6 and integrin *β*6 expression with clinicopathological factors is summarized in Table 1. Both IL-6 and integrin *β*6 expression were associated with poor tumor differentiation and advanced N stage and TNM stage. Moreover, IL-6 expression was also related to the tumor stage, while integrin *β*6 expression to M stage, indicating an association of IL-6 and integrin *β*6 expression with disease severity in the CRC patients.

### 3.2. IL-6 Induces Invasion of Human CRC Cells through IL-6R

**Figure 2:**
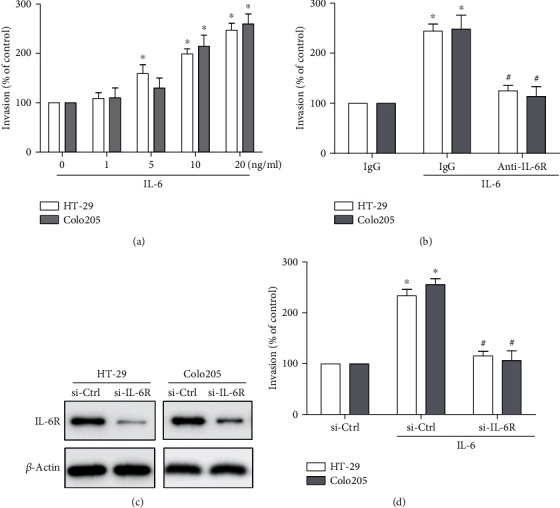
IL-6 promotes invasion of human CRC cells through IL-6R. (a) Transwell invasion assays to assess the effect of IL-6 with different concentrations on the invasion of HT-29 and Colo205 cell lines for 24 h. (b–d) Transwell invasion assays to validate the role of IL-6R in IL-6-induced cell invasion. CRC cells were pretreated for 1 h and maintained thereafter with neutralizing antibody (10 *μ*g/ml) of IL-6R (b) or pretreated for 24 h with siRNA targeting IL-6R (c, d) to inhibit IL-6R, followed by stimulation with 20 ng/ml IL-6 for 24 h. (a, b, d) Data represent the means ± SEM. *n* = 3 independent experiments. ^∗^*p* < 0.05 versus no IL-6 treatment control. ^#^*p* < 0.05 versus IL-6-treated group. (c) Data shown is representative of 3 independent experiments.

IL-6 was previously shown to increase the motility of HCT-116 CRC cells [23]. Here, the effect of IL-6 was further verified on the invasion of two other CRC cell lines HT-29 and Colo205. The invasion capability of HT-29 and Colo205 cells could be increased by IL-6 in a dose-dependent manner (Figures 2(a) and 2(b)). Inhibition of IL-6R by neutralization antibody as well as siRNA targeting IL-6R could remarkably inhibit IL-6-mediated cell invasion (Figures 2(c) and 2(d)). These data indicated that IL-6 could promote invasion via IL-6R in CRC cells.

### 3.3. Upregulation of Integrin *β*6 Involves in IL-6-Induced Invasion of CRC Cells

**Figure 3:**
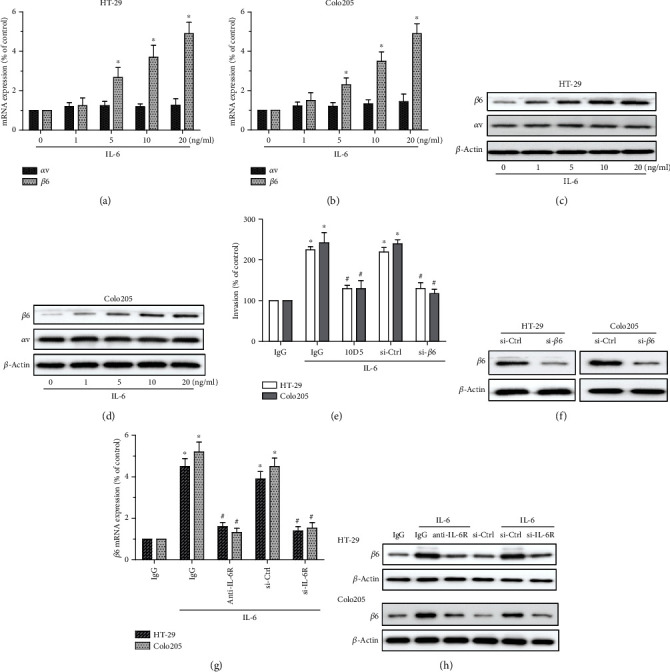
Upregulation of integrin *β*6 is critical to IL-6/IL-6R-induced invasion of CRC cells. (a, b) qPCR analysis of integrin *α*v or *β*6 gene in HT-29 (a) and Colo205 (b) cells treated with IL-6 with various doses for 24 h. (c, d) Immunoblots showing the effect of IL-6 at indicated doses on the expression of integrin *α*v or *β*6 in HT-29 (c) and Colo205 (d) cells for 24 h. (e, f) Transwell invasion assays to assess the role of integrin *β*6 in IL-6-induced invasion. CRC cells were pretreated with antibody 10D5 for 1 h and maintained thereafter to block the function of integrin *β*6 or with siRNA for 24 h to knock down *β*6, followed by stimulation with 20 ng/ml IL-6 for 24 h. (g, h) The role of IL-6R in IL-6-mediated upregulation of integrin *β*6 was validated by qPCR (g) and western blotting (h) analyses. CRC cells were pretreated with function blocking antibody against IL-6R or siRNA targeting IL-6R to inhibit IL-6R, followed by stimulation with 20 ng/ml IL-6 for 24 h. (a, b, e, g) Data represent the means ± SEM. *n* = 3 independent experiments. ^∗^*p* < 0.05 versus no IL-6 treatment control. ^#^*p* < 0.05 versus IL-6-treated group. (c, d, f, h) Data shown is representative of 3 independent experiments.

Given the positive correlation of IL-6 with integrin *β*6 in CRC samples (Figure 1(e)), and the critical role of integrin *β*6 in CRC cell migration, we hypothesized that integrin *β*6 might participate in IL-6-induced invasion of CRC cells. Based on qPCR assays, we found that the gene expression of integrin *β*6 was upregulated by IL-6 in a dose-dependent manner, while integrin *α*v was not affected (Figures 3(a) and 3(b)). Consistently, the protein level of integrin *β*6 was also increased upon IL-6 stimulation, confirming that IL-6 could upregulate the expression of integrin *β*6 (Figures 3(c) and 3(d)). In fact, as short as 15 h treatment with exogenous IL-6 was sufficient to induce integrin *β*6 transcription, indicating a potential direct regulation of integrin *β*6 by IL-6 in CRC cells (Supplementary Fig. 1a, b). In contrast, we were unable to detect the expression of integrin *β*6 in NCM460 cells in the absence or presence of exogenous IL-6 (data not shown). Furthermore, the role of integrin *β*6 in IL-6-mediated cell invasion was verified by loss-of-function study. Inhibition of integrin *β*6 with function blocking antibody 10D5 or siRNA could remarkably suppress IL-6-induced cell invasion (Figures 3(e) and 3(f)). In addition, inhibition of IL-6R reduced IL-6-upregulated transcription and translation levels of integrin *β*6 (Figures 3(g) and 3(h)). These results suggested the important participation of integrin *β*6 in IL-6/IL-6R-induced invasion of CRC cells.

### 3.4. Involvement of Integrin *β*6 in IL-6-Induced EMT

**Figure 4:**
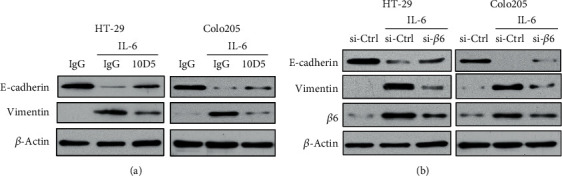
Integrin *β*6 is involved in IL-6-induced EMT of CRC cells. HT-29 and Colo205 cells were pretreated with antibody 10D5 (a) for 1 h and maintained thereafter to block the function of integrin *β*6 or with siRNA (b) for 24 h to knock down integrin *β*6, followed by stimulation with IL-6 (20 ng/ml) for 24 h. The role of integrin *β*6 in IL-6-mediated expression of EMT markers E-cadherin and Vimentin was assessed by the western blotting assay. Data shown is representative of 3 independent experiments.

EMT is well known for its pathological role in tumor invasion and metastasis, and IL-6 has been shown to induce EMT in a paracrine manner in a variety of types of carcinoma including bladder [24] and pancreatic cancers [25]. Here, we show that in addition to cell invasion, IL-6 could also promote EMT of CRC cells (Figures 4(a) and 4(b)). Since IL-6 could upregulate integrin *β*6, the cytoplasmic domain of which is critical to EMT [26]. We therefore examined whether integrin *β*6 was involved in IL-6-mediated EMT in CRC cells. Inhibition of integrin *β*6 could inhibit the suppression of E-cadherin and the induction of Vimentin mediated by IL-6 in both HT-29 and Colo205 cells (Figures 4(a) and 4(b)), thus reversing IL-6-induced EMT.

### 3.5. STAT-3 Signaling Pathway Participates in IL-6-Mediated Cell Invasion and Integrin *β*6 Upregulation

**Figure 5:**
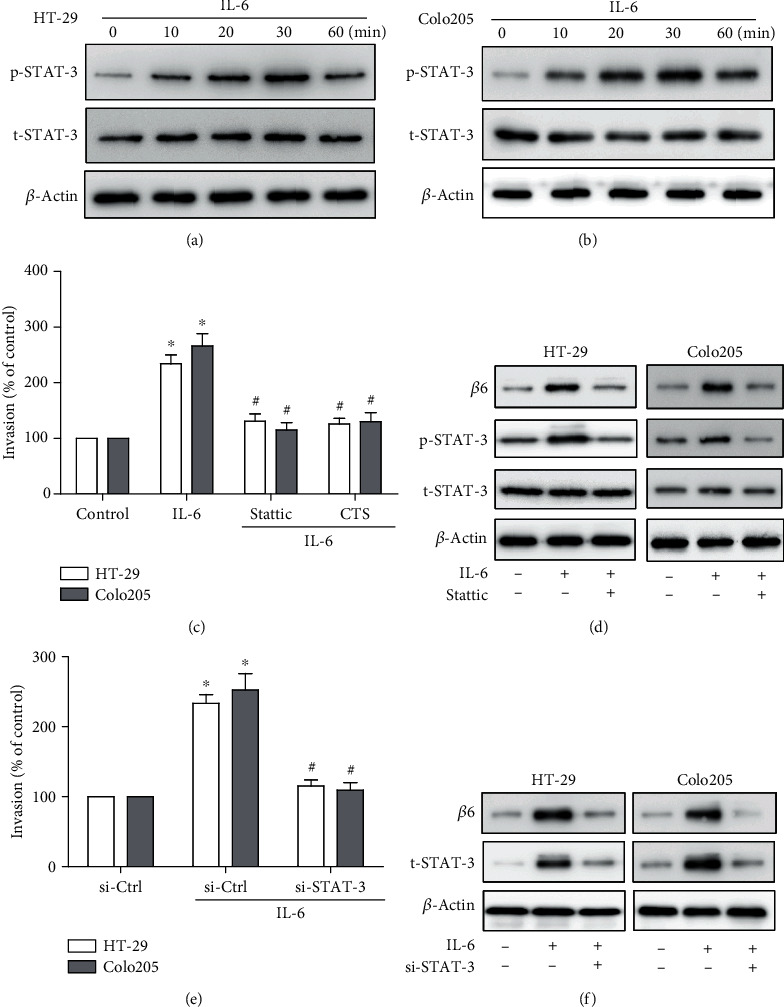
STAT-3 pathway is involved in IL-6-mediated cell invasion and upregulation of integrin *β*6. (a, b) Immunoblots showing the effect of IL-6 (20 ng/ml) with indicated treatment intervals on the expression of phospho-STAT-3 (Tyr705) (p-STAT-3) and total-STAT-3 (t-STAT-3) in HT-29 (a) and Colo205 (b) cells. (c, e) Transwell invasion assay showing the role of the STAT-3 pathway in IL-6-induced invasion. HT-29 and Colo205 cells were pretreated with STAT-3 specific inhibitor Stattic or CTS (5 *μ*M) (c) for 1 h or with siRNA (e) for 24 h to knock down STAT-3, followed by stimulation with IL-6 (20 ng/ml) for 24 h. Data represent the means ± SEM. *n* = 3 independent experiments. ^∗^*p* < 0.05 versus no IL-6 treatment control. ^#^*p* < 0.05 versus IL-6-treated group. (d, f) Immunoblots showing the role of the STAT-3 pathway in IL-6-mediated upregulation of integrin *β*6. HT-29 and Colo205 cells were pretreated with STAT-3 specific inhibitor Stattic (5 *μ*M) (d) or siRNA (f) before the treatment with IL-6 (20 ng/ml) for 24 h, and the expression of integrin *β*6 was detected. (a, b, d, f) Data shown is representative of 3 independent experiments.

The STAT-3 signaling pathway plays a prominent role in diverse cellular behaviors mediated by IL-6, including tumor cell survival, invasion, and metastasis [27]. We therefore investigated whether the STAT-3 pathway was involved in IL-6-mediated cell invasion and upregulation of integrin *β*6 in CRC cells. Upon stimulation with 20 ng/ml of IL-6, the STAT-3 pathway became activated at Tyr705, with the phosphorylation reaching the highest level at 30 min (Figures 5(a) and 5(b)), suggesting that the activation of the STAT-3 pathway by IL-6 was transient. Besides, two specific STAT-3 inhibitors were utilized to determine the role of the STAT-3 pathway in IL-6-induced invasion and upregulation of integrin *β*6. Pretreatment of cells with STAT-3 inhibitors Stattic or CTS could reduce IL-6-mediated cell invasion (Figure 5(c)). Moreover, addition of Stattic could significantly reverse IL-6-induced expression of integrin *β*6 (Figure 5(d)). Consistently, suppression of STAT-3 by siRNA could not only reduce the invasion capability but also inhibit the upregulated expression of integrin *β*6 by stimulation of IL-6 in CRC cells (Figures 5(e) and 5(f)). In contrast, while IL-6 could also expectedly activate ERK/MAPK and PI3K pathways in HT-29 cells, ERK/MAPK inhibitor U0126 could only inhibit IL-6-induced expression of integrin *β*6 in a small degree while PI3K inhibitor LY294002 had no effect (Supplementary Fig. 2a, b), indicating that MAPK and PI3K pathways were dispensable in IL-6-induced integrin *β*6 upregulation. Collectively, these data indicated that the STAT-3 signaling pathway was involved in IL-6-induced invasion and expression of integrin *β*6 in CRC cells.

### 3.6. Fibroblast-Derived IL-6 Promotes the Invasion and Upregulation of Integrin *β*6 in CRC Cells

**Figure 6:**
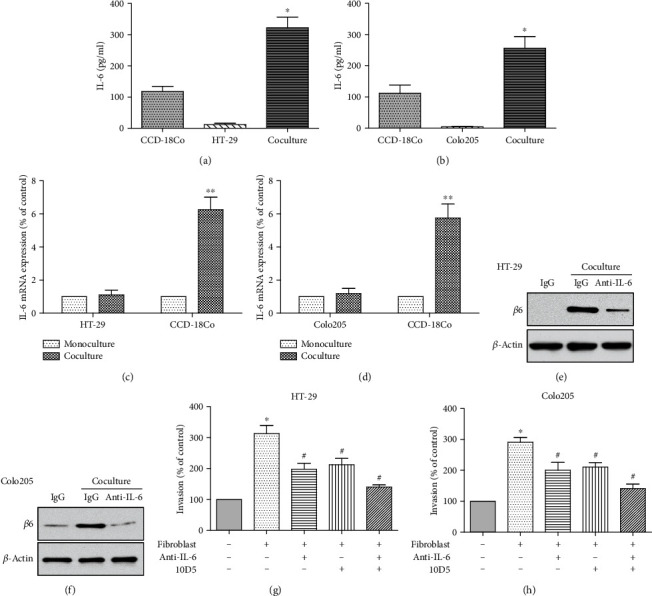
Fibroblast-derived IL-6 promotes the invasion and integrin *β*6 upregulation in CRC cells. (a, b) ELISA assay shows IL-6 secretion from the coculture system. HT-29 (a) and Colo205 (b) cells increased the production of IL-6 from CCD-18Co cells. *n* = 3 independent experiments. ^∗^*p* < 0.05 versus monoculture control. (c, d) qPCR analysis of integrin *β*6 gene in HT-29 (c) and Colo205 (d) cells as well as CCD-18Co cells under coculture compared to monoculture. Data represent the means ± SEM. *n* = 3 independent experiments. ^∗∗^*p* < 0.01 versus CCD-18Co in monoculture control. (e, f) Immunoblots showing the role of IL-6 from the coculture system in the upregulation of integrin *β*6 in CRC cells. The protein level of integrin *β*6 in HT-29 (e) and Colo205 (f) cells was increased after coculture with CCD-18Co cells and antagonized by neutralizing antibody against IL-6. Data shown is representative of 3 independent experiments. (g, h) Transwell invasion assay showing the role of IL-6 and integrin *β*6 in the enhanced invasion by coculture of CRC cells with CCD-18Co cells. Both function blocking antibodies against IL-6 and integrin *β*6 partially suppressed the coculture system-mediated invasion of HT-29 (g) and Colo205 (h) cells, and combined inhibition demonstrated the most significant abrogation. *n* = 3 independent experiments. ^∗^*p* < 0.05 versus monoculture control. ^#^*p* < 0.05 versus no antibody treatment coculture group.

To further investigate the crosstalk between cancer cells and TME, the indirect coculture assay was utilized, which showed that both HT-29 and Colo205 cells increased IL-6 secretion from fibroblasts (Figures 6(a) and 6(b)), and the mRNA level of IL-6 was predominantly induced in fibroblasts rather than CRC cell lines (Figures 6(c) and 6(d)), confirming that CRC cells could augment the production of IL-6 from the fibroblasts. Concomitantly, we also determined the effect of IL-6 secreted by fibroblasts on the expression of integrin *β*6 in CRC cells. The cancer cells in the coculture system demonstrated an upregulation of integrin *β*6, which could be antagonized via neutralizing IL-6 antibody (Figures 6(e) and 6(f)). Moreover, both IL-6 antibody and integrin *β*6 antibody inhibited the enhanced CRC cell invasion when cocultured with fibroblasts, with the combination of both function blocking antibodies presenting the most significant inhibition (Figures 6(g) and 6(h)). In contrast, integrin *β*6 expression of NCM460 cells was undetectable under coculture with fibroblasts (data not shown). Taken together, these data confirmed the role of IL-6 from TME in the promotion of tumor invasion through upregulation of integrin *β*6 in CRC cells.

## 4. Discussion

The prognosis of CRC patients with recurrent or metastatic disease remains poor, as the treatment at later stages is always an obstacle. Since tumor progression is driven not only by dysregulation of genes in cancer cells but also by various kinds of stromal cells, it has become a research hotspot to consider the paracrine crosstalk between cancer cells and TME so as to develop novel treatment strategies combining anticancer and antistroma therapies [28].

Regarding the role of TME in a tumor, the major IL-6-producing cancer-associated fibroblasts (CAFs) promote cancer stemness and induce an immune adaptive inflammatory response, thus favoring CRC progression [29]. It is well known that CAFs facilitate malignancy by secreted factors and components of ECM. In accordance, our current study shows that IL-6 secretion from fibroblasts is much more abundant than that from CRC cells, but on the other hand, CRC cells augment IL-6 secretion from fibroblasts that in return facilitates the invasion of cancer cells, forming a positive mutual enhancement between CRC and TME. The bidirectional intercellular communications participate in response to environmental cues and are necessary in regard to tumor development. Besides direct cell-cell contact, cancer cells can activate fibroblasts to initiate an inflammatory response, promoting the expression of IL-6 [30]. Multiple growth factors secreted by cancer cells, such as transforming growth factor-beta (TGF-*β*) [19] and platelet-derived growth factors [31], induce the transformation of fibroblasts. Furthermore, tumor-derived extracellular vesicles (EVs), including exosomes, are involved in tumor-fibroblast communication by transferring principal factors. EVs contain functional cellular components, such as proteins and microRNAs, which not only promote the differentiation of fibroblast into CAF but also induce chemokine secretion from fibroblasts in both proximal surrounding and distal sites [32]. In addition, tumor-derived exosomal effects in fibroblasts are also influenced by the aggressiveness of cancer cells. The molecules encapsulated in EVs derived from cancer cells with high-metastatic property contribute to an appropriate TME creation for tumor metastasis [33]. Further investigations are still needed to uncover the specific mechanisms of CRC cells to promote IL-6 secretion from fibroblasts. In addition to fibroblast-derived IL-6, we verify that exogenous IL-6 induces CRC cell invasion via IL-6R, consistent with previous study that anti-IL-6R antibody suppresses angiogenesis and inhibits the interaction between tumor and stroma [34].

As an indicator of aggressiveness, integrin *β*6 promotes EMT [35] and metastasis of CRC cells to the liver [36]. For the underlying mechanism, our study shows that IL-6/IL-6R induces the mRNA and protein levels of integrin *β*6 in a concentration-dependent manner. Besides, IL-6 is sufficient to induce integrin *β*6 transcription in a short time frame of stimulation, indicating the existence of a direct regulation. What is more important, integrin *β*6 is critical to IL-6-induced EMT and aggressiveness as inhibition of integrin *β*6 by function blocking antibody or siRNA could reverse the facilitation of invasion mediated by IL-6 in CRC cells. In contrast, we were unable to detect integrin *β*6 in NCM460 cells under exogenous IL-6 stimulation or coculture with fibroblasts, indicating that IL-6-dependent upregulation of integrin *β*6 is a feature of CRC cells rather than normal colon epithelial cells. This is consistent with the fact that integrin *β*6 is barely expressed in normal epithelia but becomes significantly upregulated, acting as a tumor promoter, in the context of malignancy. Liang et al. [22] showed that IL-6 and integrin *β*6 expression could be used as a predictor of poor overall survival of CRC; here, we not only validate the positive relationship between the expression of IL-6 and integrin *β*6 besides their association with disease severity of CRC patients in another single-center study but also show for the first time that IL-6 upregulates integrin *β*6 expression. In addition to classic signaling, transsignaling also plays an important role in the TME to recruit tumor-associated stromal cells [37]; therefore, further study is needed to investigate whether the IL-6 transsignaling pathway is involved in the process here. It should be noted that antagonizing IL-6 by neutralizing antibody in the coculture system partially inhibits invasion and integrin *β*6 expression of CRC cells, indicating that TME may secrete other growth factors to nourish and enhance CRC aggressiveness [38]. In fact, in regard to the crosstalk of integrin *β*6 with paracrine signals, the SDF-1/CXCR4 axis is able to induce directional migration and liver metastasis of CRC cells by upregulating integrin *α*v*β*6 [39]. On the other hand, integrin *α*v*β*6 of CRC cells activates fibroblasts via TGF-*β* signaling followed by increased production of SDF-1 from CAFs [19]. One important characteristic of IL-6 is the persistent activation in the whole process of inflammatory bowel disease and CRC, while the upregulations of other cytokines like TGF-*β*1, IL-10, and IL-23 only show up primarily during CRC development [38]. Therefore, even though the interaction between cancer cells and TME is dynamic and complicated, we speculate that IL-6 already starts to play an important role during the early stages of inflammatory bowel disease and CRC, thus sustaining CRC progression all the time till the rise of other paracrine signals as well as integrin pathways. Most anticancer therapies are currently developed to specifically target cancer cells, but the tumor stroma can promote the resistance of cancer cells to such therapies, eventually leading to fatal recurrence and metastasis. Our work now moves forward a further step in this complex crosstalk between CRC cells and TME, showing the mutually promotive roles of IL-6 and integrin *β*6 in CRC progression.

The discovery of TME-derived IL-6-mediated signaling pathways is helpful for further understanding the mechanism underlying CRC metastasis, which may contribute to the development of therapeutic strategies. Among the signaling pathways, STAT-3 is the main transactivator downstream of IL-6 signaling, and IL-6 is involved in the activation of oncogenic STAT-3 in many kinds of cancer [40]. Therefore, we investigated whether IL-6-induced upregulation of integrin *β*6 is mediated by STAT-3 signaling. IL-6 stimulation leads to phosphorylation of STAT-3 (Tyr 705), and inhibition of STAT-3 by specific inhibitors or siRNA abrogates IL-6-induced invasion and integrin *β*6 upregulation, indicating the participation of this classical signaling cascade in response to IL-6. Aberrant activities of STAT-3 contribute to malignancy and thus are a potential therapeutic target for CRC. It should be noted that in our experiments, exogenous IL-6 activates STAT-3 transiently as the phosphorylated level of STAT-3 reached the peak within 30 min upon exogenous IL-6 stimulation, probably due to suppressor of cytokine signaling 3 (SOCS3). The activation of IL-6 signaling cascade can induce SOCS3 expression, which forms a negative feedback loop to terminate the STAT-3 signaling pathway in turn, leading to a basal state for the cells. Both epigenetic suppression of SOCS3 [41] and the paracrine signals in the TME [42] may contribute to the sustaining stimulus for STAT-3 activation in multiple forms of cancer. Nevertheless, the transient STAT-3 activation here is still efficient to induce integrin *β*6 upregulation in CRC cells. The combination of STAT-3 and integrin *β*6 expression has been reported to have a better prognostic performance than either alone in gallbladder carcinoma [43]. STAT-3 has been shown to be involved in promoting integrin *β*6 transcription in oral squamous cell carcinoma [44] and prostate cancer cells [45]. Moreover, Niu et al. [46] recently characterized the region located at the core promoter of integrin *β*6 and demonstrated the binding sites for transcription factor STAT-3. The present study now shows that STAT-3 is a critical transcription factor involved in IL-6-induced upregulation of integrin *β*6 in CRC cells. In our prior study, we found that the MAPK/Ets-1 signaling pathway was also involved in the dysregulation of integrin *β*6 [47]; however, through pathway inhibitors, we found that ERK/MAPK and PI3K pathways were dispensable in IL-6-induced integrin *β*6 expression, suggesting the preferred role of the STAT-3 pathway in IL-6-mediated CRC progression. Our future work will further characterize the mechanisms of the crosstalk between CRC and TME so as to provide valuable candidates for preventing or treating CRC progression.

## 5. Conclusions

In summary, our results reveal a novel link between paracrine signals originating from TME and increased integrin *β*6 level of CRC. We for the first time discover that integrin *β*6 becomes upregulated upon IL-6 stimulation through the IL-6R/STAT-3 pathway, and integrin *β*6 plays a vital role in IL-6-induced EMT and invasion of CRC cells. These findings have potential therapeutic implications for targeted therapy. Since integrin *β*6 participates in the paracrine crosstalk between the TME and CRC cells, combined inhibition of IL-6 signaling along with integrin *β*6-targeted strategies may indicate new directions for antitumor strategies for CRC.

## Figures and Tables

**Table 1:** Association of IL-6 and integrin *β*6 expression with clinicopathological factors in CRC cases.

Clinicopathological factors	*n*	IL-6 expression		*p*	Integrin *β*6 expression		*p*
		Low	High		Negative	Positive	
Gender				0.458			0.660
Male	83	40	43		49	34	
Female	72	39	33		45	27	
Age (years)				0.443			0.746
<60	66	36	30		41	25	
≥60	89	43	46		53	36	
Differentiation				0.009			<0.001
Well	53	35	18		41	12	
Moderate	59	29	30		40	19	
Poor	43	15	28		13	30	
Tumor stage				0.013			0.083
T1-T2	91	54	37		50	41	
T3-T4	64	25	39		44	20	
N stage				<0.001			<0.001
N0	62	49	13		47	15	
N1	58	21	37		35	23	
N2	35	9	26		12	23	
M stage				0.436			0.006
M0	132	69	63		86	46	
M1	23	10	13		8	15	
TNM stage				0.029			0.002
I-II	75	45	30		55	20	
III-IV	80	34	46		39	41	

## Data Availability

The raw data related to this paper may be requested from the corresponding author.

## Abbreviations

CRC: Colorectal cancer
TME: Tumor microenvironment
EMT: Epithelial-mesenchymal transition
IL-6: Interleukin-6
IL-6R: IL-6 receptor
ECM: Extracellular matrix
MMP: Matrix metalloproteinase
CAF: Cancer-associated fibroblast
IHC: Immunohistochemistry
ELISA: Enzyme-linked immunosorbent assay
TGF-*β*: Transforming growth factor-beta
EVs: Extracellular vesicles
SOCS3: Suppressor of cytokine signaling 3.

## Supplementary Materials

Supplementary Materials Supplementary Fig. 1: effect of IL-6 on the transcription of integrin *β*6 of CRC cells. qPCR analysis of integrin *β*6 gene in HT-29 (a) and Colo205 (b) cells treated with IL-6 (20 ng/ml) for different period intervals. Data represent the means ± SEM. *n* = 3 independent experiments. ^∗^*p* < 0.05 versus no IL-6 treatment control. Supplementary Fig. 2: ERK/MAPK and PI3K pathways are dispensable in IL-6-induced integrin *β*6 expression. HT-29 cells were pretreated with ERK/MAPK inhibitor U0126 (20 *μ*M) (a) or PI3K inhibitor LY294002 (20 *μ*M) (b) for 1 h before the treatment with IL-6 (20 ng/ml) for 24 h, and the expression of integrin *β*6 was detected. Data shown is representative of 3 independent experiments.
