# Supplementary material for: Interleukin-6 Promotes Epithelial-Mesenchymal Transition and Cell Invasion through Integrin β6 Upregulation in Colorectal Cancer
Source: Oxid Med Cell Longev. 2020 Aug 13;2020:8032187. doi: 10.1155/2020/8032187 (PMC7443035; doi:10.1155/2020/8032187)
Supplement: Supplementary Materials — Supplementary Fig. 1: effect of IL-6 on the transcription of integrin β6 of CRC cells. qPCR analysis of integrin β6 gene in HT-29 (a) and Colo205 (b) cells treated with IL-6 (20 ng/ml) for different period intervals. Data represent the means ± SEM. n = 3 independent experiments. ∗p < 0.05 versus no IL-6 treatment control. Supplementary Fig. 2: ERK/MAPK and PI3K pathways are dispensable in IL-6-induced integrin β6 expression. HT-29 cells were pretreated with ERK/MAPK inhibitor U0126 (20 μM) (a) or PI3K inhibitor LY294002 (20 μM) (b) for 1 h before the treatment with IL-6 (20 ng/ml) for 24 h, and the expression of integrin β6 was detected. Data shown is representative of 3 independent experiments. [file 8032187.f1.zip › mat.8032187.v3.pdf]

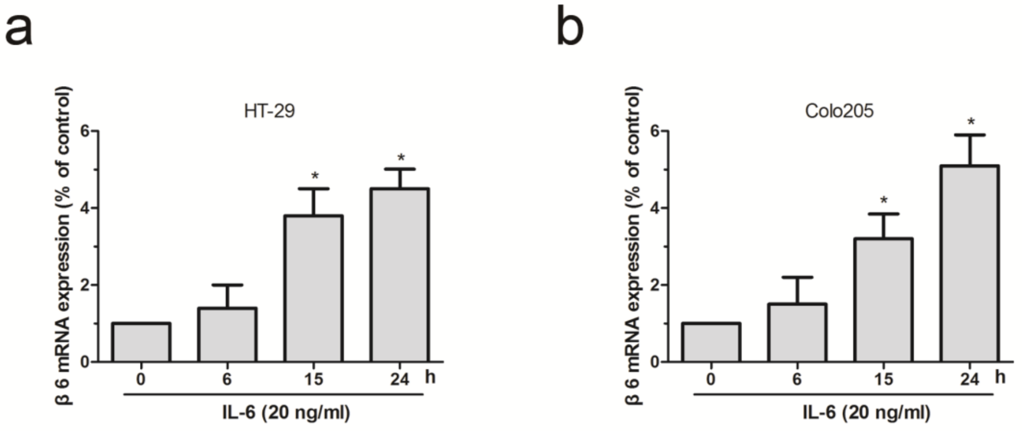

Supplementary Fig. 1 Effect of IL-6 on the transcription of integrin β6 of CRC cells. qPCR analysis of integrin β6 gene in HT-29 (**a**) and Colo205 (**b**) cells treated by IL-6 (20 ng/ml) for different period intervals. Data represent the means ± SEM. n=3 independent experiments. $^{*}p<0.05$ versus no IL-6 treated control.

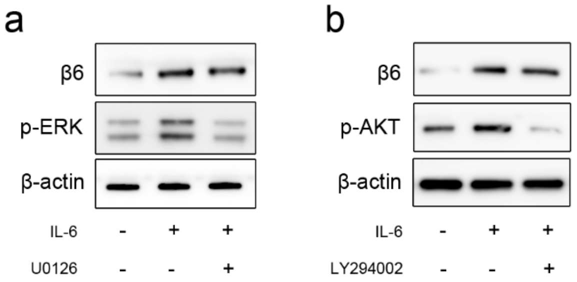

Supplementary Fig. 2 ERK/MAPK and PI3K pathways are dispensable in IL-6-induced integrin β6 expression. HT-29 cells were pretreated with ERK/MAPK inhibitor U0126 (20 μM) (**a**), or PI3K inhibitor LY294002 (20 μM) (**b**) for 1 h before the treatment with IL-6 (20 ng/ml) for 24 h, and the expression of integrin β6 was detected. Data shown is representative of 3 independent experiments.
